# Public health round-up

**DOI:** 10.2471/BLT.24.010524

**Published:** 2024-05-01

**Sudan crisis one year in**

A child receives vitamin A drops during an integrated health, nutrition, water, sanitation and hygiene campaign implemented by the United Nations Children’s Fund (UNICEF) and partners at Alnahda gathering point, River Nile State, Sudan. The state is receiving a significant proportion of the 8.1 million people fleeing their homes as a result of the ongoing civil war in the country, which marked its first anniversary on 15 April.

**Figure:**
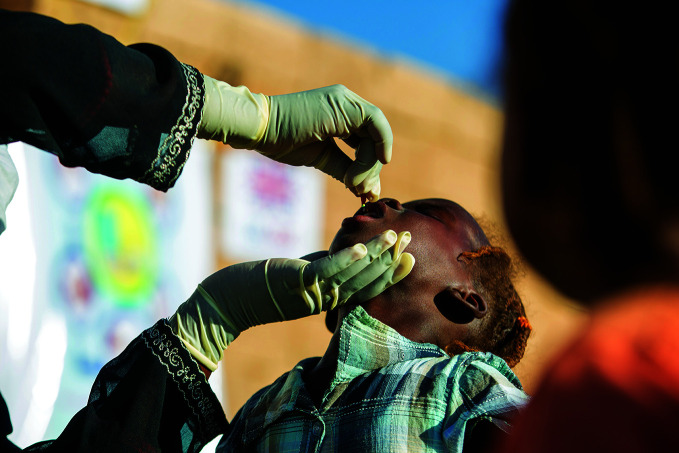
UNICEF/Ahmed Elfatih Mohamdeen

## Sudan crisis

The civil war in Sudan passed the one-year mark on 15 April. The nationwide conflict has created the world’s largest humanitarian crisis with some 24.8 million people assessed to require humanitarian assistance in 2024, 9 million more than in 2023.

The number of people displaced by the conflict continues to increase, with 8.1 million people assessed to have fled their homes. Approximately 6.3 million of those people are displaced within Sudan while another 1.8 million people have fled abroad.

The health system is collapsing, especially in hard-to-reach areas, with health facilities destroyed, looted or struggling with acute shortages of staff, medicines, vaccines, equipment and supplies. Some 30 million people are assessed to have no access to health-care services.

As of 15 April, only 17% of the 2.7 billion United States dollars called for under the humanitarian needs and response plan (which serves as the basis for implementing and monitoring the collective response of the humanitarian community) had been funded, and the World Health Organization (WHO) called for the international community to increase its contributions.

https://bit.ly/4az6OwK

## Gaza’s health system in ruins

A WHO-led multi-agency mission assessed Al-Shifa Hospital in north Gaza on 5 April to evaluate the extent of destruction following recent fighting there.

The mission, conducted in partnership with several United Nations agencies and the acting hospital director, revealed severe devastation. Restoring even minimal functionality in the short term was judged unlikely, and would require substantial efforts to clear the grounds of unexploded ordnance to allow partners to bring in equipment and supplies.

The ongoing conflict has left only 10 of the 36 main hospitals that used to serve over 2 million Gazans somewhat functional, with severe limitations on the types of services they can deliver.

In a 6 April statement, WHO emphasized the urgent need for the protection of health-care facilities, personnel and civilians, as well as the establishment of safe and efficient aid corridors to address the escalating humanitarian crisis in Gaza.

https://bit.ly/3TLiJjM

## Hepatitis rising

The number of deaths attributed to viral hepatitis is on the rise. According to the 2024 global hepatitis report published by WHO on 9 April, an estimated 1.3 million people die from viral hepatitis infections annually, on par with deaths associated with tuberculosis.

Released at the World Hepatitis Summit, the report includes new data from 187 countries which indicate that that the estimated number of deaths from viral hepatitis increased from 1.1 million in 2019 to 1.3 million in 2022. Of these, 83% were caused by hepatitis B, and 17% by hepatitis C.

The report highlights the fact that despite better tools for diagnosis and treatment, and decreasing product prices, testing and treatment coverage rates have stalled.

“This report paints a troubling picture: despite progress globally in preventing hepatitis infections, deaths are rising because far too few people with hepatitis are being diagnosed and treated,” said WHO Director-General Tedros Adhanom Ghebreyesus. “WHO is committed to supporting countries to use all the tools at their disposal […] to save lives and turn this trend around.”

https://bit.ly/3JbZAT7

## Polio emergency maintained for tenth year

The thirty-eighth meeting of the Emergency Committee under the International Health Regulations (2005) on the international spread of poliovirus reviewed data on wild poliovirus (WPV1) and circulating vaccine-derived polioviruses (cVDPV), and unanimously agreed that the risk of international spread of poliovirus still remains a Public Health Emergency of International Concern (PHEIC).

Meeting on 20 March, the committee recommended the extension of the PHEIC for a further three months. The WHO Director-General accepted the recommendation.

The Committee acknowledged the concerns regarding the lengthy duration of the polio PHEIC, which has been in place since May 2014, and stressed the importance of exploring alternative measures.

https://bit.ly/4aqqhiR

## Nigeria launches new meningitis vaccine

Nigeria launched the world's first vaccination campaign using Men5CV, a new WHO-endorsed vaccine.

Men5CV offers protection against the five major strains of the meningococcus bacteria (A, C, W, Y and X), as opposed to the vaccine used in much of the African Region, which is only effective against the A strain.

Launched on 12 April, the initiative was supported by Gavi, the Vaccine Alliance (Gavi), which manages the global meningitis vaccine reserve and aids low-income countries in routine immunization.

Nigeria experienced a significant increase in meningitis cases last year, including a severe outbreak of *Neisseria meningitidis* serogroup C, with 1,742 suspected cases, 101 confirmed cases, and 153 deaths in seven states from October 2023 to March 2024.

A large-scale vaccination drive was conducted 25–28 March 2024, targeting over a million people aged 1–29 years in the affected areas.

https://bit.ly/3U1BWO6

## Cholera diagnostics initiative

The arrival of rapid diagnostic test kits for cholera in Malawi on 5 April marked the start of a global programme set to distribute over 1.2 million tests to 14 cholera-prone countries in the coming months.

The largest-ever global deployment of its kind, the initiative aims to enhance outbreak detection and response by bolstering routine surveillance and testing capacity.

Countries severely affected by cholera outbreaks, such as Ethiopia, Somalia, Syrian Arab Republic and Zambia, will receive the kits, enabling them to promptly identify probable cholera cases and monitor trends for future preventive programmes.

The initiative is funded and coordinated by Gavi and led by UNICEF in collaboration with WHO and the Global Task Force on Cholera Control.

Cholera outbreaks have surged globally since 2021, underscoring the urgent need for improved diagnostic capabilities and preventive measures along with broader efforts to address cholera's root causes, mainly safe water and sanitation.

https://bit.ly/3vAwj1z

**Cover photo**

A community health worker brings his 7-year-old son to Musovu Health Post in Bugesera District, Rwanda.

**Figure:**
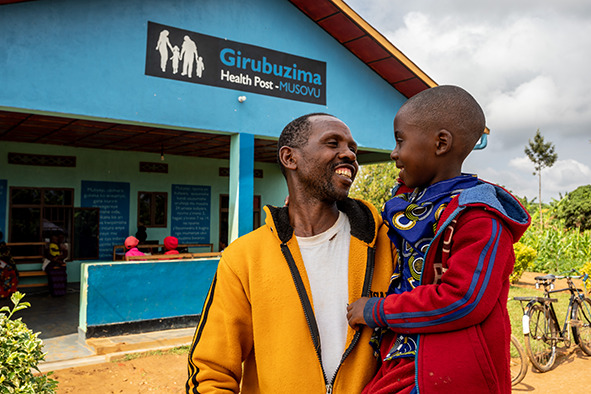
WHO/Isaac Rudakubana

## A new AI health avatar

WHO introduced SARAH (Smart AI Resource Assistant for Health), a new digital health avatar that uses generative artificial intelligence (AI) to offer empathetic and personalized health information.

Launched on 2 April, SARAH uses technology developed and supported by Soul Machines, an AI avatar development company, that allows for real-time, accurate and empathetic communication without the restrictions of pre-set scripts or algorithms. The avatar can communicate in 8 languages and operate around the clock on any device.

SARAH’s primary role is to help people understand and manage their health better by providing reliable information on key health topics such as healthy living, mental health, and high-burden diseases such as cancer, heart disease, lung disease and diabetes.

Previous iterations of SARAH have been used to disseminate critical public health messages on topics ranging from COVID-19 to tobacco use.

https://bit.ly/3VQWoDZ

## Finalizing a global pandemic agreement

WHO Member States agreed to continue negotiations from April 29 to May 10 to finalize a global pandemic agreement. The decision followed intensive discussions during the ninth meeting of the Intergovernmental Negotiating Body (INB) which began on March 18.

Those talks focused on key issues such as financing, equitable access to medical countermeasures, and health workforce strengthening to improve readiness and response for future pandemics.

Co-chairs of the INB Bureau, Precious Matsoso and Roland Driece, highlighted the critical nature of the negotiations, underscoring the need for a consensus-based approach to prepare for and respond to future pandemics with solidarity and equity.

Member States are scheduled to consider the proposed text of the world’s first pandemic agreement for adoption at the Seventy-seventh World Health Assembly which starts on 27 May 2024.

https://bit.ly/3VPmqaT

**Looking ahead**

14–15 May 2024. Launch of the WHO SPECS 2030 initiative. Geneva, Switzerland https://bit.ly/3PXIBIe

23–24 May. The Global Partners Meeting for Nursing and Midwifery. Geneva, Switzerland. https://bit.ly/3TZ1Pyq

27 May–1 June. Seventy-seventh World Health Assembly. Geneva, Switzerland. https://bit.ly/4aJVM7s

